# Combined effects of temperature, salinity, and diet simulating upwelling and nonupwelling seasons alter life‐history characteristics of a tropical invertebrate

**DOI:** 10.1002/ece3.5873

**Published:** 2019-12-12

**Authors:** Alejandra Camargo‐Cely, Rachel Collin

**Affiliations:** ^1^ Smithsonian Tropical Research Institute Balboa Ancon Panama; ^2^ Present address: Department of Biology University of Puerto Rico Río Piedras Puerto Rico

**Keywords:** Bay of Panama, *Crepidula* cf. *marginalis*, multifactorial experiment, multiple stressors, upwelling

## Abstract

Upwelling is known to affect the ecology and life history of temperate nearshore organisms, and these effects are thought to be mediated by changes in temperature and food supply. However, little information is available for tropical systems. To understand how changes in the intensity of upwelling might impact marine invertebrates, we tested how factorial combinations of temperature, salinity, and phytoplankton availability affected growth and reproduction of a common intertidal snail, *Crepidula* cf. *marginalis*. We used temperatures typical of nonupwelling (29°C), moderate (26°C) and severe (23°C) upwelling, salinities typical of nonupwelling (30 ppt) and upwelling (34 ppt) and a good diet (*Isochrysis*) and a better diet (*Isochrysis* and *Tetraselmis*) as a proxy for increased productivity during upwelling. Overall, temperature and diet had consistent effects on body size, with better food and lower temperatures promoting larger size, as well as promoting shorter time to first reproduction. Diet had the largest effects on clutch size, with clutch size increasing with better diet. Temperature had the largest effect on offspring size and the frequency of discarded broods; offspring size decreased with increasing temperature and the frequency of discarded broods also decreased with increasing temperatures. We found no significant 3rd order interactions and few significant strong 2nd order interactions, which have often been found in similar experimental studies using stressful treatments. For this tropical slipper limpet, the effect of higher food and cooler temperatures during upwelling appears to be positive, promoting higher growth rates, larger clutch sizes, and larger offspring size suggesting that both factors likely play an important role underlying reproductive responses to upwelling. Climatic changes, like El Niño, which suppress upwelling in the Bay of Panama, appear likely to negatively impact this species.

## Graphical abstract

To understand how they interact to effect life histories, we raised tropical snails (*Crepidula* cf. *marginalis*) under a factorial combination of diet, temperature, and salinity. The results show that a combination of lower temperatures and higher food availability results in increased growth, larger broods, and larger hatching sizes. *C*. cf. *marginalis*, therefore, performs better under conditions similar to upwelling compared to those similar to nonupwelling conditions experienced in the Bay of Panama. This effect is largely mediated through the increased body size under higher food and cooler temperatures.

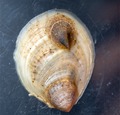

## 1 INTRODUCTION

Multiple stressor experiments are becoming increasingly common as researchers focus effort on understanding and predicting the impacts of global change (Gunderson, Armstrong, & Stillman, 2016; Todgham & Stillman, 2013). Despite predictions that environmental change is likely to involve the concomitant change in many important environmental conditions at the same time or in close sequence (Bopp et al., 2013), studies including more than two factors are still relatively rare, especially in marine systems (Przeslawski, Byrne, & Mellin, 2015). Many experiments maximize the chances of finding detectable effects by using large but justified differences in conditions, with treatments often representing extreme conditions or falling outside conditions currently experienced by the study organism. However, multifactorial studies focused on conditions that are currently experienced and which are not obviously stressful can also provide insight into potential responses to near‐term climate change. They are especially valuable in understanding if factors interact synergistically or antagonistically at ecologically relevant values (Crain, Kroeker, & Halpern, 2008; Folt, Chen, Moore, & Burnaford, 1999). Here we take this approach, using a multifactorial experiment to determine how environmental conditions typical of two different seasons impact growth and reproduction individually and in combination.

The early impacts of climate change include subtle changes, including alterations in the timing and duration of seasons (Sparks & Menzel, 2002). In temperate systems where reproduction and biological productivity are largely linked to the summer, the impacts of such changes may involve a lengthening of the growing season as well as changes in the timing of spring reproductive events (Sparks & Menzel, 2002). In tropical systems switches between monsoonal and nonmonsoonal conditions, which are driven by changes in the position of the intertropical convergence zone (ITCZ), may dominate the seasonal cycles of many organisms (e.g., Gaonkar et al., 2012; Pillai & Subramoniam, 1984; Wai et al., 2012). Such patterns are already impacted by El Niño‐Southern Oscillation (ENSO; the cycle of sea surface temperatures in the central and eastern Pacific, which has global climate consequences through shifts in atmospheric circulation; see Glynn, 1988) events which, for example, increase the length of the dry season in Panama. In terrestrial eco‐systems, this results in increased drought and fruit production by trees in the local forest (Wright, Carrasco, Calderon, & Paton, 1999), and in marine systems in a reduction in intensity and duration of seasonal upwelling along the Pacific coast (Glynn, 1984, 1988). Climate models predict a narrowing (Byrne & Schneider, 2016) and a latitudinal shift (Chen, Langenbrunner, & Randerson, 2018; Seo, Kang, & Merlis, 2017) of the ITCZ, which could result in changes in the distribution and duration of the wet and dry seasons in the equatorial tropics, and therefore alter the duration of the upwelling season along the Pacific coast of Central America.

Although tropical marine systems generally lack the strong seasonality found in temperate systems, the Bay of Panama, one of three centers of upwelling along the Central American coast, has distinct, well‐defined seasons. The upwelling season (January–May) is characterized by cool, nutrient‐rich surface water, with high salinity. The rainy season (May–December) is characterized by the absence of upwelling and corresponding higher temperatures, lower salinity, and less nutrient availability (D'Croz & O'Dea, 2007). Biological responses to upwelling in the Bay of Panama include high phytoplankton productivity (D'Croz, Del Rosario, & Gómez, 1991; D'Croz & Robertson, 1997; Smayda, 1963), hydromedusae blooms, (Miglietta, Rossi, & Collin, 2008), and reduced reproduction of some fishes (Robertson, 1990) and invertebrates (Collin & Ochoa, 2016; Lessios, 1981). During El Niño years, the typical effects of upwelling are reduced or suppressed, as winds are reduced and the oceanic thermocline deepens. In strong La Niña years, the thermocline shoals, upwelling can be particularly strong and water temperatures can plummet (Glynn, 1984, 1988; Glynn, Mate, Baker, & Calderón, 2001; Robertson & Collin, 2015).

To understand how changes in the duration and intensity of the upwelling season might impact marine invertebrates, we tested how factorial combinations of temperature, salinity, and phytoplankton availability affected growth and reproduction of a common intertidal snail. These factors co‐vary in nature but may act independently or synergistically, and this approach allowed us to disentangle these effects. We used the slipper limpet *Crepidula* cf. *marginalis* a common intertidal calyptraeid gastropod, which reproduces year‐round in the Bay of Panama (Collin & Ochoa, 2016). Calyptraeids are protandrous sequential hermaphrodites, and species of *Crepidula* are emerging as model systems for studies of lophotrochozoan development (Henry, Collin, & Perry, 2010). The filter‐feeding, benthic adults produce small eggs packaged into capsules which are brooded by the mothers. Planktotrophic larvae hatch after 8–10 days (Collin, 2012). Females produce multiple broods in a year, and species in the family are extremely abundant and diverse in this region, suggesting they are well‐suited to the prevailing environmental conditions.

## 2 MATERIALS AND METHODS

*Crepidula* cf. *marginalis* were collected at Playa Venado (8.892°N, 79.597°W) near the town of Veracruz on the Pacific coast of the Bay of Panama in November 2017. Collecting was covered by a permit issued by the Panamanian Ministry of Environment (SE/A‐73‐17). Animals were gently removed from under small rocks in the mid to high intertidal. We collected 360 small animals in their male or juvenile stages (<7 mm shell length) by selecting only small snails that were found on top of females. They were immediately transported submerged in seawater to the Naos Island laboratories of the Smithsonian Tropical Research Institute (STRI) in Panama City.

Each individual was placed separately in a 350‐ml plastic cup, water was changed 3 times a week, and the animals were fed five times a week. After 2 weeks of acclimation to the laboratory, animals were assigned haphazardly to one of a fully factorial combination of three treatments: diet (good and better), temperature (23, 26, and 29°C), and salinity (30 and 34 ppt), resulting in twelve experimental treatments. These treatments were chosen as a reflection of upwelling and nonupwelling conditions experienced in the Bay of Panama. The selected values for temperature and salinity characterize conditions that are normally experienced by these animals in the field and which vary seasonally (Collin & Ochoa, 2016; D'Croz & O'Dea, 2007). During the dry season (January–May), upwelling results in ocean surface water which is cool (~20–25°C), has high salinity (~34 ppt), is rich in nutrients, and supports plankton blooms. During the rainy season (May–December), the absence of upwelling results in surface waters with higher temperatures (~28–29°C), lower salinity (~30 ppt), and less nutrient and phytoplankton availability (D'Croz & O'Dea, 2007). Daily measurements of the seawater at the shore, directly in front of the Naos marine laboratory at the seawater intake taken over 15 years, show that monthly average temperatures during February and March (the coldest months) can range from 21.5 to 26.2°C depending on the year. To capture these differences between strong and weak upwelling, we used two cooler temperatures to compare to the nonupwelling temperatures. Realized temperatures in the treatments were 29.3°C (*SD* = 0.01), 25.6°C (*SD* = 0.22) and 22.8°C (*SD* = 0.13) as measured once a day in a water‐filled cup using an Omega High Accuracy Digital Thermometer.

Slipper snails feed on phytoplankton and other suspended material (Beninger, Decottignies, Guiheneuf, Barillé, & Rincé, 2007; Decottignies, Beninger, Rincé, Robins, & Riera, 2007; Shumway et al., 2014) and can be reared for their entire life cycle and reproduce successfully on an exclusive diet of *Isochrysis galbana* (Collin, 2012; Collin & Ochoa, 2015). We provided 20 × 10^6^ cells/ml daily of *Isochrysis galbana* for snails in the good diet treatment (following the high food treatment of Mérot & Collin, 2012), and for the better diet, we provide the same amount of *Isochrysis* and 3.33 × 10^6^ cells/ml of *Tetraselmis* sp. As the cell volume of *Tetraselmis* is six times that of *Isochysis*, this provided not only a mix of food, but double the cell volume. A mixed diet of *Isochrysis* and *Tetraselmis* has been shown to be a better diet for gastropod larvae in aquaculture (Aranda, Lucas, Brule, Salguero, & Rendon, 1989).

To implement the temperature treatments, each animal was placed in an individual cup and 120 cups were assigned to each of three incubators, one at each experimental temperature. The snails were randomly allocated to the four combinations of diet and salinity and distributed randomly within the incubator. Temperature was monitored during the experiment to ensure no abnormal fluctuations were experienced. When we changed the water, the temperature and salinity were measured using a Professional Series Pro 2030 YSI. If the salinity of our raw seawater supply was too low, salinity was increased by partially freezing containers with seawater and removing the ice to increase the salinity without altering its other qualities. This saltier water was added to regular seawater to adjust it to the desired salinity. Salinity was reduced by adding distilled water.

**Figure 1:**
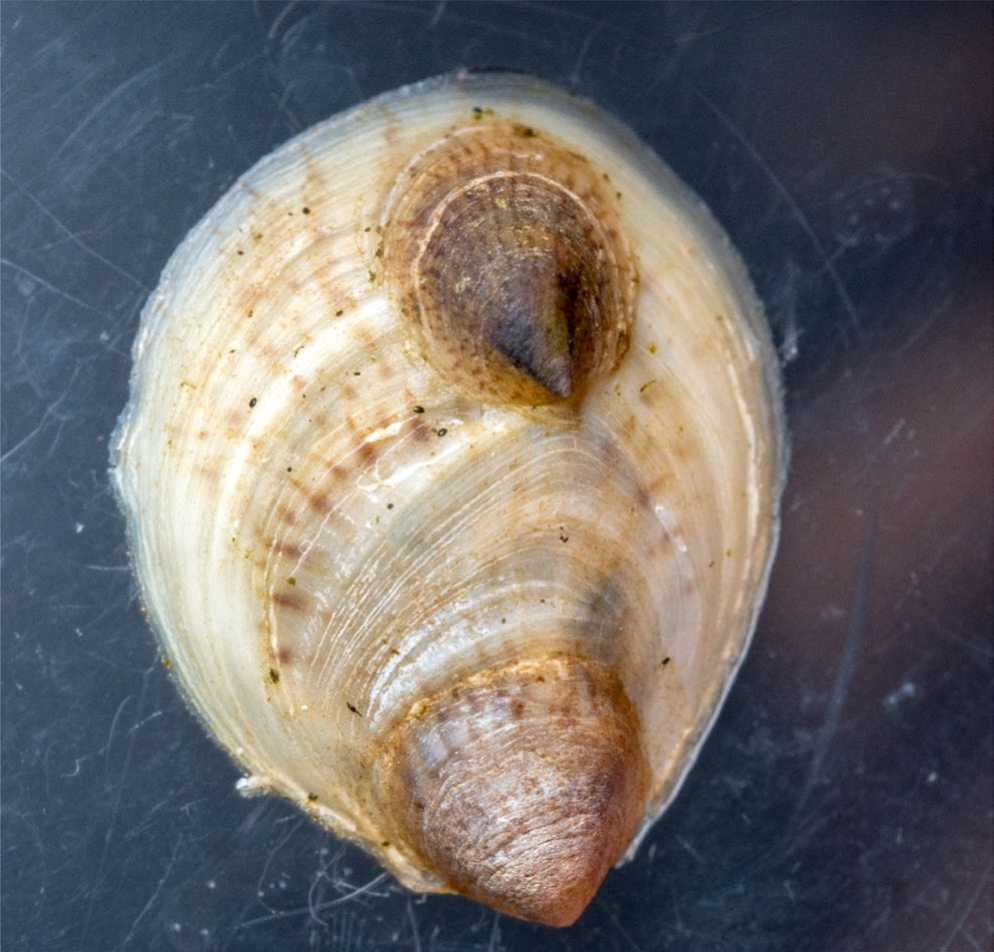
A pair of *Crepidula* cf. *marginalis*. A small male is usually positioned on the shell of the larger female

To document differences in growth rate between the treatments, snails were measured prior to allocation to the experimental treatments, and every 2 weeks thereafter for 8 weeks. At this point, most of the animals were females and growth had slowed significantly. We added a single small male to each cup (Figure 1) and stopped measuring the growth rate. To determine how the treatments impacted reproductive success, we measured clutch size and hatching size for each female (following Collin, 2012; Collin & Salazar, 2010). Clutch size was calculated as the number of egg capsules in a brood times the average number of eggs in five capsules. Hatching size was measured for twenty ethanol‐preserved larvae per brood which were collected the day they hatched naturally. The larval shell length was measured using ImageJ v. 1.51s (Abramoff, Maelhaes, & Ram, 2004) to measure the Feret diameter (the longest diameter) of the shell. Female size was measured when broods or hatchlings were collected. We also recorded the size at first reproduction, the time to first reproduction, the time to hatching of one brood for each female, and the number of broods that were aborted by the female. At the end of the experiment, the females were sacrificed and shell length and dry weight were measured.

The effect of each factor and their interactions on growth rate were examined using a fully factorial repeated measures analysis of variance (RM ANOVA) using the MANOVA function in the JMP statistical package (version 12; SAS Inc.). Clutch size and final dry body weight were both standardized by maternal shell length before being analyzed. In all cases, stepwise removal of nonsignificant interactions employing the forward AICc criterion was used to simplify the model. If the stepwise removal function in JMP retained a first, second, or third order effect, we used that as the upper limit of the effects in a subsequent factorial model ANOVA and we retained all effects of that order. We studied the residuals for normality or approximate normality to ensure that the assumptions of ANOVA were adequately met (Zuur, Ieno, & Elphick, 2010). Variances were sufficiently homogenous, as the ratio of the largest to the smallest variance did not exceed 4, and the sample sizes were all similar (Tabachnik & Fidell, 2012; Zuur et al., 2010). Only the number of days to laying did not adequately fit the assumptions of ANOVA. Transformations failed to improve the fit, and therefore, a nonparametric test was used with these data. During the experiment, we noticed that some females discarded broods, moving them out from under their neck, and ejecting them into the cup. We used contingency analysis and a *χ* ^2^ test to determine if the likelihood that a female discarded a brood differed between the treatments.

## 3 RESULTS

Overall, temperature and diet both had significant effects on aspects of the growth or reproduction of *Crepidula* cf. *marginalis*. Temperature and diet had consistent effects on body size, with better food and lower temperatures promoting larger size, as well as promoting shorter time to first reproduction. Diet had the largest effect on clutch size, with clutch size increasing with better diet. Temperature had the largest effect on offspring size and the frequency of discarded broods; size decreased with increasing temperature and the frequency of discarded broods also decreased with increasing temperatures.

### 3.1 Growth rates and body size

**Figure 2:**
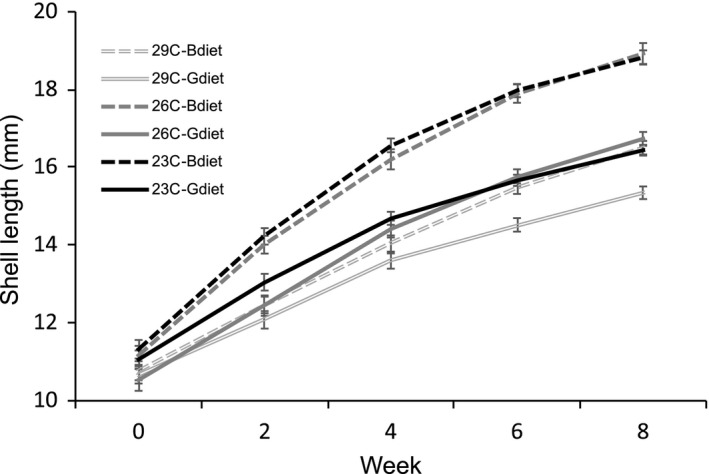
Effect of temperature and diet on shell length measured over the first eight weeks of the experiment. Error bars represent the standard errors

At the initiation of the experiment, we found no difference in shell length between the different treatments (For all factors in the fully factorial model: ANOVA *p* > .05; *N* = 349; *R* ^2^ = .04). Repeated measures ANOVA with a fully factorial model showed significant effects of temperature and diet, and of the interaction between temperature and diet on size (Table 1; Figure 2). Animals raised at 23°C or 26°C grew significantly larger than those raised at 29°C. The better diet resulted in larger animals at each temperature compared to those on the good diet. For example, animals fed a better diet at 29°C grew at a similar rate to animals raised at 23°C or 26°C with a good diet.

**Table 1:** Results from the between subjects tests in the repeated measures ANOVA examining the effects of the three environmental factors on the growth of *C*. cf. *marginalis*

Factors	*df*	Exact *F*	*p*
Temperature	2	40.23	**<.0001**
Salinity	1	1.00	.32
Diet	1	78.83	**<.0001**
Temperature × salinity	2	2.79	.063
Temperature × diet	2	5.41	**.005**
Salinity × diet	1	1.18	.28

Note

*N* = 335. Statistically significant results are highlighted in bold.

Size at first reproduction varied significantly between treatments (Table 2). Animals raised with a better diet were slightly, but significantly larger at their first brood than those raised on the good diet (Better diet: mean = 19.85 mm; *SE* = 0.17. Good diet: mean = 18.22 mm; *SE* = 0.18). The size at first brood decreased significantly with increasing temperature and differed significantly between all three temperature treatments. Mean size at 29°C was 17.6 mm (*SE* = 0.21), mean size at 26°C was 19.09 mm (*SE* = 0.21), and mean size at 23°C was 20.39 mm (*SE* = 0.21). There was not interaction between diet and temperature. Final size at the end of the experiment increased by less than 1 mm from these sizes and showed the same pattern of significance (results not shown).

**Table 2:** ANOVA table of the effects of the three environmental variables on the size that a female first produced a brood

Source	*df*	*F* ratio	*p*
Temperature	2		**<.0001**
Salinity	1	1.89	.17
Diet	1	43.44	**<.0001**

Note

*N* = 156; *R* ^2^ = .45. Statistically significant results are highlighted in bold.

The time to first reproduction was inversely related to temperature. It took 96 (*SE* = 0.27) days at 29°C, 101 (*SE* = 0.28) days at 26°C and 109 (*SE* = 0.33) days at 23°C (Wilcoxon nonparametric pair‐wise comparisons show significant differences between 23°C and 29°C but not between 26°C and the other two temperatures). Because males were added once the size at sex change was attained in each treatment, males were added later to the low‐food treatment and we could not statistically test for a significant effect of diet on time to first clutch. Because the time from the addition of the male to the first brood did not differ between the diet treatments, our results show that animals on the better diet lay their first clutch ~23 days sooner than those on a good diet.

**Figure 3:**
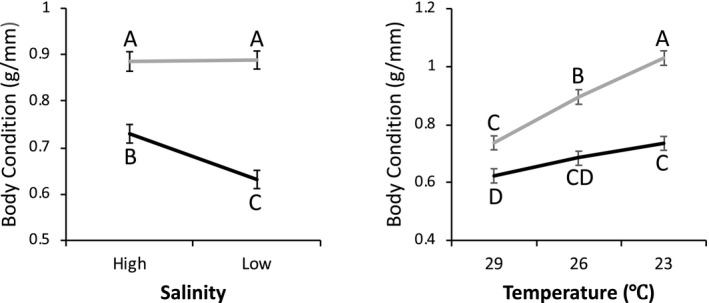
The effects of interactions between diet and salinity, and diet and temperature on body condition (dry weight/shell length) measured at the end of the experiment. Error bars represent the standard errors. Letters above the points represent the results of the post hoc Tukey HSD test. The black line is for the good diet and the gray line is for better diet

Final dry body weight standardized by shell length reflected body condition. This was significantly affected by two different significant interactions involving diet (Table 3; Figure 3), after four outliers were removed. The significant interaction salinity and diet illuminated by a post hoc Tukey HSD test showed a significantly higher body condition with a better diet than with a good diet. With a better diet, salinity did not have an affect on body condition, but under a good diet body condition was lower at low salinities. The other significant interaction was between diet and temperature. Animals raised with a better diet had a better body condition at all temperatures than they did under a good diet. Under the better diet, body condition increases with decreasing temperature. Under a good diet, the same trend is evident, but it is not significant.

**Table 3:** ANOVA table of the effects of the three environmental variables on final dry body weight standardized by shell length

Source	*df*	*F* ratio	*p*
Temperature	2	34.53	**<.0001**
Salinity	1	5.67	**.018**
Diet	1	106.18	**<.0001**
Temperature × salinity	2	2.64	.07
Temperature × diet	2	6.85	**.001**
Salinity × diet	1	6.40	**.012**

Note

*N* = 317 *R* ^2^ = .40. Statistically significant results are highlighted in bold.

### 3.2 Reproductive success

**Figure 4:**
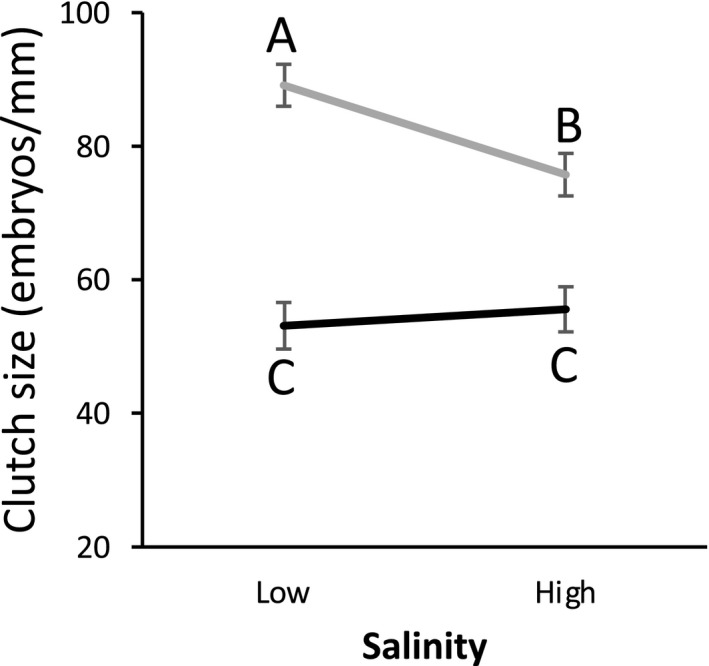
Effects of the interaction between diet and salinity on standardized clutch size (clutch size/shell length). Error bars represent the standard errors. Letters above the points represent the results of the post hoc Tukey HSD test. The black line is for the good diet and the gray line is for better diet

**Figure 5:**
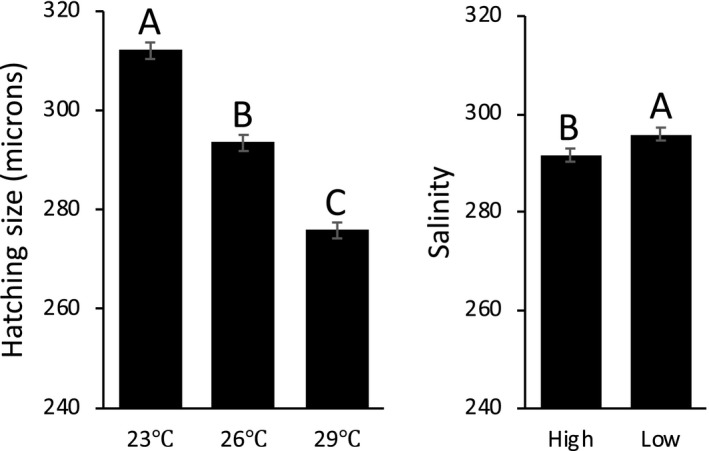
Effects of temperature and salinity on hatching size. Error bars represent the standard errors. Letters above the bars represent the results of the post hoc Tukey HSD test

For clutch size, the stepwise removal retained up to second‐order factors in the ANOVA (Table 4), but the only significant interaction was salinity by diet. A post hoc Tukey HSD test showed no effect of salinity under the good diet, but larger clutch size at low salinity than at high salinity when raised under the better diet (Figure 4). A better diet resulted in significantly larger clutch sizes (Table 4; Figure 4). Average hatching size was analyzed with an ANOVA with only direct effects (Table 5; Figure 5). Hatching size significantly decreased with increasing temperature, and hatching size was larger at lower salinity (Figure 5).

**Table 4:** ANOVA table of the effects of the three environmental variables on total clutch size standardized by female length

Source	*df*	*F* ratio	*p*
Temperature	2	3.48	**.03** a
Salinity	1	2.73	.10
Diet	1	75.50	**<.0001**
Temperature × salinity	2	1.48	.22
Temperature × diet	2	0.81	.45
Salinity × diet	1	5.76	**.017**

Note

*N* = 287; *R* ^2^ = .24. Statistically significant results are highlighted in bold.

a Tukey post hoc test did not detect a significant difference between the three temperatures.

**Table 5:** ANOVA table of the effects of the three environmental variables on hatching size

Source	*df*	*F* ratio	*p*
Temperature	2	122.26	**<.0001**
Salinity	1	5.18	**.024**
Diet	1	0.42	.52

Note

*N* = 275, *R* ^2^ = .48. Statistically significant results are highlighted in bold.

Both diet and temperature had significant direct effects on time to hatch. Time to hatch is slightly shorter with a better diet at 9.2 (*SE* = 0.12) days compared to 9.7 (*SE* = 0.13) days with a good diet (Table 6). Time to hatch decreases significantly with increasing temperature averaging 10.6 days at 23°C, 9.2 days at 26°C, and 8. 5 days at 29°C.

**Table 6:** ANOVA table of the effects of the three environmental variables on time to hatch

Source	*df*	*F* ratio	*p*
Temperature	2	48.28	**<.0001**
Salinity	1	0.12	.73
Diet	1	9.37	**.0024**

Note

*N* = 279, *R* ^2^ = .27. Statistically significant results are highlighted in bold.

The 12‐state variable “treatment” marginally affected the likelihood a female would discard at least one brood during the experiment (Likelihood ratio *χ* ^2^ = 19.38; *p* = .055; *N* = 308; *df* = 11). Post hoc tests for each variable individually showed that only temperature had a significant (i.e., *p* < .05) effect on the proportion of females that discarded broods. At high temperatures, 19% of females discarded at least one brood, at medium temperature 37% discarded broods, and at low temperatures 41% discarded at least one brood (Likelihood ratio *χ* ^2^ = 13.71; *p* = .001; *N* = 308; *df* = 2).

## 4 DISCUSSION

Our experimental approach to understanding the effects of normal seasonal variation in environmental conditions highlights the importance of examining, not just multiple factors but multiple life‐history response variables as well. This is most vividly illustrated by the effects of temperature on growth and reproduction (Tables 7 and 8). Most species living in the Bay of Panama are tropical, their ranges extend through areas of the tropical eastern Pacific which do not experience upwelling while in the Bay of Panama upwelling significantly impacts water temperatures for 4 months of the year. The tropical fishes in the area reduce reproduction during upwelling, and it is thought that this is due to cold stress as these species are adapted to uniformly warm temperatures (Robertson, 1990; Robertson & Collin, 2015). It was therefore surprising that *Crepidula* cf. *marginalis* seemed to perform more poorly in terms of growth rate and offspring size (two commonly used metrics of performance and fitness) at 29°C than at the lower temperatures. In contrast to the smaller body size and smaller offspring size at 29°C, however, animals grown at 29°C reproduced sooner and at smaller sizes, had broods that hatched more quickly, and were less likely to discard a brood. Taken together, in addition to reduced performance at warmer temperatures, there appears to be a plastic shift in life‐history strategy at 29°C compared to 23 and 26°C.

**Table 7:** Summary of the significant effects

	Growth	Size at first brood	Time to first brood	Final size	Final weight	Clutch size	Hatching size	Discarded broods
Temperature	↓	↓	↓	↓	↓	—	↓	↓
Salinity	—	—		—	X	X	↑	—
Diet	↓	↑	↓	↑	↑	↑	—	—

Note

Arrows showing the direction of the effect, with down arrows indicating a negative relationship (e.g., growth decreases with increasing temperature) and with an up arrow indicating a positive relationship. “X” indicates a role in an interaction without a clear directional effect. “—” indicates no significant role in explaining variance in that particular features.

**Table 8:** Summary of life table values with conditions representing weak and strong upwelling conditions, as well as nonupwelling conditions highlighted

Temperature (°C)	Salinity (ppt)	Food	Size at 8 weeks (mm)	Mean clutch size (*SE*)	Mean hatching size (μm) (*SE*)	Mean dry weight (g) (*SE*)	Mean days to hatch (*SE*)	Mean broods discarded
23	30	Good	16.41 (0.21)	867 (77)	320.63 (1.25)	0.0140 (0.0007)	11.25 (0.43)	0.88 (0.33)
23	30	Better	18.83 (0.25)	1,753 (133)	312.31 (0.79)	0.0230 (0.0009)	1,043 (0.31)	0.69 (0.22)
23	34	Good	17.25 (0.22)	1,045 (80)	306.26 (0.75)	0.0152 (0.0007)	10.22 (0.39)	0.58 (0.17)
**23**	**34**	**Better**	**19.51** (0.24)	**1,697** (151)	**311.30** (0.95)	**0.0217** (0.0009)	**10.71** (0.29)	**0.81** (0.21)
26	30	Good	16.48 (0.17)	920 (78)	297.14 (1.06)	0.0121 (0.0006)	9.63 (0.33)	0.75 (0.19)
26	30	Better	19.01 (0.27)	1,797 (129)	297.00 (1.08)	0.0191 (0.0007)	8.92 (025)	0.45 (0.13)
26	34	Good	16.40 (0.22)	961 (102)	295.85 (0.96)	0.0131 (0.0008)	9.44 (0.37)	0.33 (0.14)
**26**	**34**	**Better**	**18.64** (0.25)	**1,391** (103)	**288.81** (0.65)	**0.0177** (0.0009)	**8.85** (0.26)	**0.44** (0.12)
***29***	***30***	***Good***	***15.38*** (0.24)	***1,109*** (86)	***276.46*** (1.04)	***0.0097*** (0.0005)	***8.71*** (0.26)	***0.34*** (0.15)
29	30	Better	16.24 (0.21)	1,746 (117)	277.70 (0.77)	0.0136 (0.0006)	8.04 (0.26)	0.19 (0.09)
29	34	Good	15.31 (0.23)	1,077.6 (66)	272.63 (0.88)	0.0125 (0.0004)	9.12 (0.32)	0.24 (0.10)
29	34	Better	16.77 (0.28)	1,390 (129)	276.49 (0.86)	0.0148 (0.0007)	8.25 (0.25)	0.33 (0.13)

Note

The two bold rows highlight results under conditions similar to those experienced during upwelling and the bold italic row highlights results under conditions similar to those experienced during nonupwelling. Mean values are given with the standard error in parentheses.

In contrast to the apparently contradictory results of temperature on performance, diet appeared to have a uniformly positive impact on performance, when a significant effect was detected. A better (mixed and more abundant) diet resulted in higher growth rates, larger clutch sizes, larger size at first reproduction, and a greater increase in body condition. It also appeared likely that a better diet promoted a shorter time to first reproduction. Finally, salinity had only a subtle effect on growth and reproduction, which was mediated through interactions with diet and temperature. Low salinity depressed dry body weight under lower food conditions and promoted large clutch sizes under high food conditions. These interactions do not form a coherent picture of the overall impact of different salinities, and the effects were generally small.

Examining the impact of multiple factors is as important as examining multiple responses. In the field, organisms are exposed to co‐varying environmental conditions or multiple stressors which may act individually, synergistically, or antagonistically. If strong interactions are common, the results of single factor/stressor studies could over‐ or under‐estimate the impact of these factors in the real world. Complicated interactions can also impact the efficacy of natural selection to act on performance under stressful conditions. Meta‐analyses of the limited data available for multiple stressors in marine systems have shown that as the number of stressors increases, the frequency of interactions doubles and the effects of these interactions are more frequently synergistic (Crain et al., 2008).

Our study appears to be somewhat unusual in that we did not find any significant 3‐way interactions between temperature, salinity, and diet for any of the life‐history responses we measured. In general, the main effects were strong and significant, while the two‐way interactions were marginally significant (*p* > .01). The strongest interaction was the interaction between temperature and diet on growth rate (Table 1; Figure 2) which also had a significant knock‐on effect on several of the other responses presumably via the influence of body size. In this case, the interaction appeared to be synergistic. For initial growth rate, a better diet increased growth across all three treatments but the increase was larger at the lower temperatures (Figure 2).

### 4.1 How do our results relate to field observations?

In a recent review of stressors in marine invertebrate development, Przeslawski et al. (2015) observed that in many cases laboratory experiments cannot be linked to or were not linked to field data, ground‐truthing the effects detected in the laboratory. Previous field work on *C*. cf. *marginalis* provides some comparative data for hatching size and for the propensity to brood. Although *C*. cf. *marginalis* brood all year, they are slightly more likely to have a clutch under them during the upwelling season (63% brooding) than during the nonupwelling season (52% brooding; Collin & Ochoa, 2016). This is consistent with the idea that upwelling conditions promote reproduction in the field. However, time to hatching is slower at cooler temperatures (Table 6). If the interbrood period is similar, the 20% increase in time to hatch between 29 and 23°C could result in a 10% difference in the number of animals brooding. Although offshore temperatures during upwelling in the Bay of Panama usually range from 20 to 25°C, and can dip as low as 15°C (D'Croz & O'Dea, 2007; Robertson & Collin, 2015), upwelling temperatures measured in the high part of the intertidal habitat of these snails, averaged 27°C across the entire 2014 upwelling season (Collin & Ochoa, 2016). As we found virtually no difference in time to hatch between 26 and 29°C in the laboratory, the temperature difference observed in that field study seems unlikely to be the cause of the seasonal difference in the number of animals brooding, leaving open the idea that reproduction may be more frequent during upwelling. These field observations were likely made at the higher end of temperatures experienced by the species overall, as temperatures were logged at the high intertidal edge of the species distribution, which is warmed more by emersion than lower in the species distribution. In addition, monthly average shallow (~2–4 m) water temperatures measured only 10km away were warm that year (January = 25.4°C, February = 23.4°C, March = 24.5°C; April = 25.7°C), while temperatures in 2013 were lower with monthly averages of 21.8°C in February and 23.3°C in March.

Offspring size also varies with season in the field (Collin & Ochoa, 2016). In the field, average hatching size is slightly larger during the upwelling season (275.3 μm) than during the nonupwelling season (267.4 μm; Collin & Ochoa, 2016). Hatching size increased through the upwelling season, with the largest (284.66 μm) average hatching size attained in April (Collin & Ochoa, 2016). Laboratory experiments demonstrate that egg size and hatching size generally decrease with temperature in calyptraeids (Collin, 2012; Collin & Salazar, 2010; Collin & Spangler, 2012), which appears to account for this size difference. However, it may be noteworthy that the hatching sizes reported from the field study are ~10 μm smaller than those reported in the lab at the same temperature (Table 8 and Collin, 2012). This may be due to any number of conditions that differ between laboratory and field, but may be associated with diet, as our diet did not well simulate the natural phytoplankton assemblage. Alternately, they could result from the difference between the constant temperatures experienced in the laboratory, and the fluctuating temperatures experienced in the intertidal (Collin & Ochoa, 2016). Such alternating temperatures can have synergistic effects. For example, Sanford (2002) has shown that alternating temperatures designed to simulate the periodic upwelling typical of the Oregon coast promote higher growth rates in a snail and a starfish compared to constant temperatures. It is possible that something similar could be affecting offspring size in *C*. cf. *marginalis*.

### 4.2 How does upwelling impact reproductive ecology?

Understanding the spatial and temporal patterns of adult fecundity, which shape the initial pool of larvae in the water column, is vital to fully understand the factors affecting the supply of settlers which form the focus of “supply‐side” ecology. One of the most elegant demonstrations of the importance of propagule supply (Hughes et al., 2000) showed that 72% of variation in coral recruitment among regions of the Great Barrier Reef was explained by the fecundity of adult corals in the region. Upwelling is known to have significant bottom‐up impacts on the ecology of benthic organisms (Barth et al., 2007; Broitman, Navarrete, Smith, & Gaines, 2001; Menge et al., 1997, 2003) and therefore may also significantly impact reproductive effort and therefore the supply of propagules. Data are limited, but it appears that patterns of upwelling can significantly impact gonad size and condition, as well as biosynthetic capacity for benthic invertebrates. For example, a comparison of upwelling and nonupwelling sites found that increased food availability during upwelling results in higher keyhole limpet body condition and larger gonads as well as higher biosynthetic capacity, indicated by a higher RNA: DNA ratio (Pulgar et al., 2013). Likewise, spatial variation in reproductive output of sea urchins reflects oceanographic conditions that impact nearshore nutrient flux and in turn increase adult food supply (Lester, Gaines, & Kinlan, 2007). By necessity, these field studies are correlative, but our experiments support the important role of adult diet, showing that a better diet results in higher body condition, larger sizes, and larger clutch sizes. However, in this tropical limpet the effect of higher food and cooler temperatures during upwelling appear to be additive or synergistic, with cooler temperatures also promoting higher growth rates and larger clutch sizes, suggesting that both factors likely play an important role.

Upwelling is also known to significantly impact larval dynamics and settlement in temperate systems (e.g., Ma & Grassle, 2004; Miller & Emlet, 1997; Narváez, Navarrete, Largier, & Vargas, 2006; Queiroga et al., 2007; Wing, Largier, Botsford, & Quinn, 1995). Most of these studies have been conducted in the Eckman upwelling dominated eastern boundary current systems along the West Coast of the Americas. In these systems, upwelling is most pronounced in the spring and summer, coinciding with the reproductive season of many marine invertebrates and fishes. Therefore, short‐term (day to week) changes in intensity during the upwelling season can have significant impacts on recruitment through larval supply to the juvenile habitats (Barth et al., 2007; Miller & Emlet, 1997; Wing et al., 1995). In contrast, upwelling in the Bay of Panama may coincide with a reduction in reproduction of some fishes and invertebrates (Collin & Ochoa, 2016; Lessios, 1981; Robertson, 1990). For example, spawning by three species of reef fishes is lowest but recruitment was highest during upwelling (Robertson, 1990). In contrast, in common intertidal hermit crabs, increased growth resulting in reproductive peaks appear to be caused by increased food availability during upwelling (Bertness, 1981). Finally, many intertidal invertebrates in the Bay of Panama reproduce year‐round, including several fiddler crab species (Kerr, Christy, Collin, & Guichard, 2012), the intertidal sand dollar *Melita stokes* (Dexter, 1977), and the high intertidal isopod, *Excirolana braziliensis* (Cardoso & Defeo, 2003). Unfortunately, the seasonal patterns of reproduction in many common and ecologically important invertebrates remain unknown. Such variation among species, as well as subtle trade‐offs within species, like those reported here suggest that changes in upwelling will impact the marine fauna of the Bay of Panama.

The small‐scale temporal changes in upwelling during the upwelling season may have less of an impact on recruitment in the Bay of Panama, than the differences between upwelling seasons driven by the ENSO cycles. However, it seems likely that increases in upwelling, like those seen during La Niña years, will negatively impact the reproductive success of a number of tropical specialists, while other species may remain relatively unaffected. Unfortunately, predictions of future changes in marine environmental conditions are lacking for the region and so little is known about the reproductive ecology of the inhabitants that it is too early to make any kind of predictions from these few studies. However, our experiments show that the reproductive success of species like *C*. cf. *marginalis*, which does reproduce year‐round, can be significantly impacted by changes in environmental conditions typical of those experienced seasonally in the Bay of Panama.

## 5 CONCLUSIONS

Taken together, these results show that a combination of lower temperatures and higher food availability result in increased growth, larger broods, and larger hatching sizes. *C*. cf. *marginalis*, therefore, clearly performs better under conditions similar to upwelling compared to those similar to nonupwelling conditions experienced in the Bay of Panama. This effect is largely mediated though the increased growth rate under higher food and cooler temperatures.

## CONFLICT OF INTEREST

None declared.

## AUTHOR CONTRIBUTIONS

AC‐C acquired the data, conducted preliminary analyses, and drafted an original report. RC conceived the study, conducted the statistical analyses, and drafted the final manuscript.

## Data Availability

The data acquired and analyzed for this study have been deposited in FigShare (https://doi.org/10.6084/m9.figshare.10006994).
